# Biparametric magnetic resonance imaging-based radiomics features for prediction of lymphovascular invasion in rectal cancer

**DOI:** 10.1186/s12885-023-10534-w

**Published:** 2023-01-18

**Authors:** Pengfei Tong, Danqi Sun, Guangqiang Chen, Jianming Ni, Yonggang Li

**Affiliations:** 1 Department of Radiology, Jiangnan University Medical Center, Wuxi, 214000 Jiangsu China; 2 Department of Radiology, the First Affiliated Hospital of Soochow University, Suzhou, 215006 Jiangsu China; 3 Department of Radiology, the Second Affiliated Hospital of Soochow University, Suzhou, Jiangsu China

**Keywords:** Rectal cancer, Lymphovascular invasion, Biparametric MRI, Radiomics

## Abstract

**Background:**

Preoperative assessment of lymphovascular invasion(LVI) of rectal cancer has very important clinical significance. However, accurate preoperative imaging evaluation of LVI is highly challenging because the resolution of MRI is still limited. Relatively few studies have focused on prediction of LVI of rectal cancer with the tool of radiomics, especially in patients with negative statue of MRI-based extramural vascular invasion (mrEMVI).The purpose of this study was to explore the preoperative predictive value of biparametric MRI-based radiomics features for LVI of rectal cancer in patients with the negative statue of mrEMVI.

**Methods:**

The data of 146 cases of rectal adenocarcinoma confirmed by postoperative pathology were retrospectively collected. In the cases, 38 had positive status of LVI. All patients were examined by MRI before the operation. The biparametric MRI protocols included T2-weighted imaging (T2WI) and diffusion-weighted imaging (DWI). We used whole-volume three-dimensional method and two feature selection methods, minimum redundancy maximum relevance (mRMR) and least absolute shrinkage and selection operator (LASSO), to extract and select the features. Logistics regression was used to construct models. The area under the receiver operating characteristic curve (AUC) and DeLong’s test were used to evaluate the diagnostic performance of the radiomics based on T2WI and DWI and the combined models.

**Results:**

Radiomics models based on T2WI and DWI had good predictive performance for LVI of rectal cancer in both the training cohort and the validation cohort. The AUCs of the T2WI model were 0.87 and 0.87, and the AUCs of the DWI model were 0.94 and 0.92. The combined model was better than the T2WI model, with AUCs of 0.97 and 0.95. The predictive performance of the DWI model was comparable to that of the combined model.

**Conclusions:**

The radiomics model based on biparametric MRI, especially DWI, had good predictive value for LVI of rectal cancer. This model has the potential to facilitate the clinical recognition of LVI in rectal cancer preoperatively.

**Supplementary Information:**

The online version contains supplementary material available at 10.1186/s12885-023-10534-w.

## Background

Colorectal cancer is a common malignant tumor of the digestive tract. The global morbidity and mortality rates associated with colorectal cancer have been increasing in recent years. This cancer is the second most common cause of cancer-related death, and rectal cancer accounts for one-third of the incidence of colorectal cancer [1, 2]. At present, surgical resection combined with neoadjuvant chemoradiotherapy is the main treatment for rectal cancer [2–4]. Previous studies have shown that invasion of tumor cells into the microcirculation around the rectum increases the risk of spread and metastasis of rectal cancer. Vascular invasion of rectal cancer is an independent risk factor for postoperative tumor recurrence and low tumor-free survival [5, 6]. A recent study showed that lymphovascular invasion(LVI), rather than the traditionally believed depth of tumor invasion, is even a key risk factor for early rectal cancer metastasis [7]. Therefore, preoperative assessment of LVI of rectal cancer, including identification of small lymphatic and vascular invasion, has very important clinical significance for predicting postoperative recurrence, metastasis, and tumor-free survival of rectal cancer.

High-resolution magnetic resonance imaging (MRI) is the main imaging method for rectal cancer, and it plays a very important role in assessment of the tumor in the preoperative and postoperative periods. Biparametric MRI, including T2-weighted imaging (T2WI) and diffusion-weighted imaging (DWI), has demonstrated reliable results in the detection and staging of rectal cancer [8]. High-resolution MRI shows good performance in the demonstration of larger vessels. In the detection of extramural vascular invasion(EMVI),MRI has moderate sensitivity and high specificity. However, MRI cannot definitively depict vessels with a diameter of < 3 mm, including micro-arteriovenous and lymphatic vessels [9, 10]. Therefore, accurate preoperative imaging evaluation of LVI of rectal cancer is highly challenging.

Radiomics relies on algorithm models to extract quantitative imaging features that cannot be captured by visual inspection. The selected radiomics features can be used to establish predictive model related to disease diagnosis and treatment combined with clinical and pathological data. At present, radiomics as an emergent imaging analysis is widely used in the imaging studies of tumor diagnosis, post-treatment, and prognosis evaluation [11–13]. In the study of rectal cancer, research has shown that radiomics has good predictive value for nodal metastasis and extramural venous invasion as well as evaluation of neoadjuvant radiotherapy and chemotherapy in patients with rectal cancer [14]. However, relatively few studies have focused on preoperative prediction of LVI of rectal cancer especially in patients with the negative statue of MRI-based extramural vascular invasion(mrEMVI).

The present study was performed to explore the preoperative predictive value and role of biparametric MRI-based radiomic features for LVI of rectal cancer in patients with the negative statue of mrEMVI.

## Materials and methods

### Patients

**Fig. 1:**
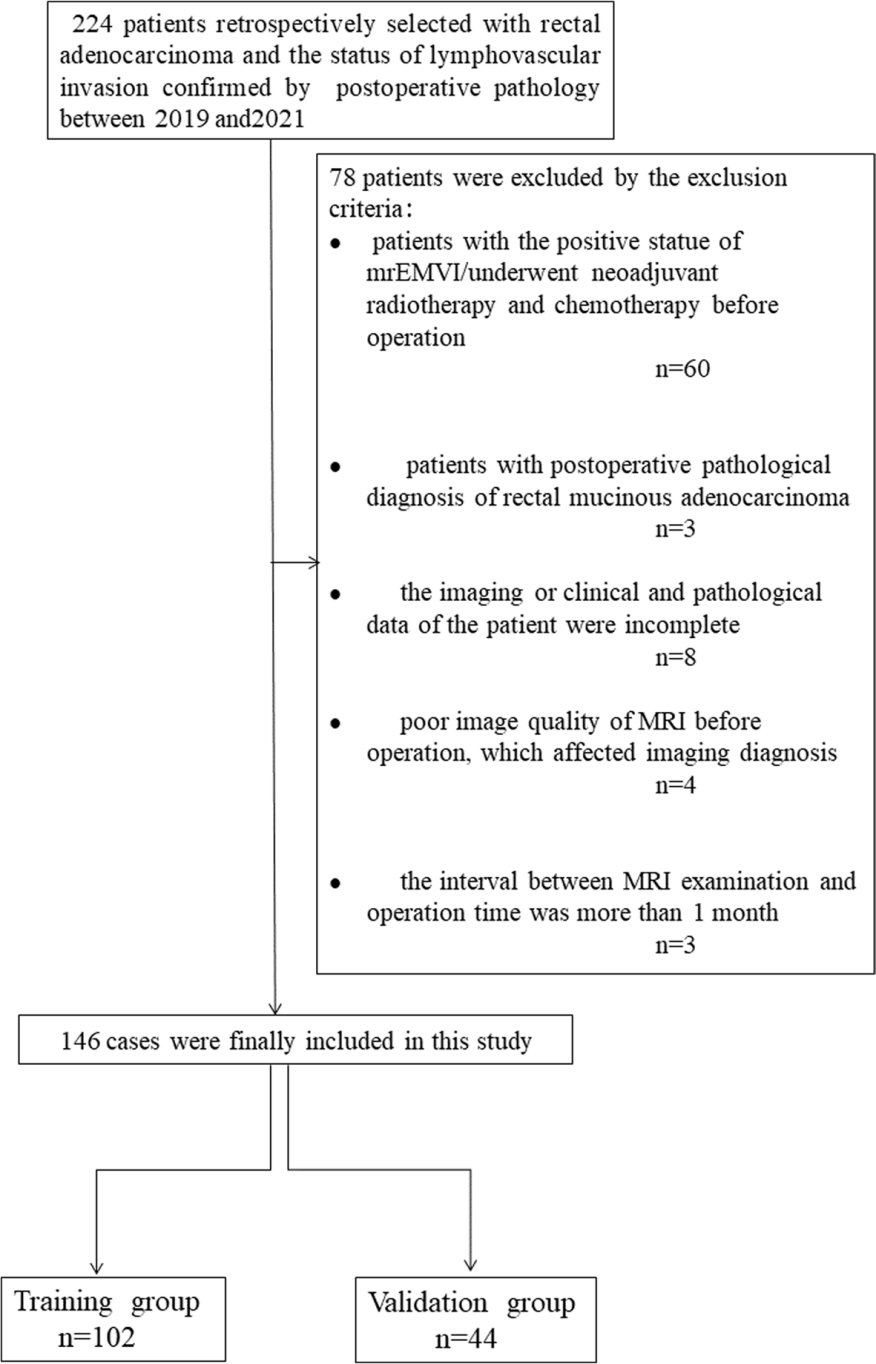
Flow chart of patient selection in this study

Cases of rectal adenocarcinoma confirmed by surgery and postoperative pathology were retrospectively collected from 2019 to 2021. The inclusion criteria were rectal adenocarcinoma confirmed by surgery and postoperative pathology, examination by noncontrast-enhanced MRI before the operation, and confirmation of the status of LVI by postoperative pathology. The exclusion criteria were patients with treatment before the operation; patients with the positive statue of mrEMVI; incomplete imaging or clinical and pathological data; poor image quality of MRI before the operation, affecting the imaging diagnosis; and a > 1-month interval between the MRI examination and operation. Finally, 146 cases were included. A study flow chart is shown in Fig. 1.

### Image acquisition

All patients underwent preoperative MRI examinations with a Siemens Skyra 3.0 T system (Siemens Healthineers, Erlangen, Germany), and intestinal cleaning and preparation were performed before the examination. The biparametric MRI protocols included T2WI and DWI. Images from each sequence were obtained in the oblique coronal, oblique axial, and sagittal planes. The oblique axial plane was determined by the perpendicular axis of the tumor, and the oblique coronal plane was parallel to the tumor axis or anal canal depending on the location of the tumor. The details of the scanning sequences are shown in Table 1.

**Table 1:** Rectal MRI scanning parameters

	T2WI	DWI
Parameter		b = 0,1000 s/mm^2^
Anatomical planes	oblique axial oblique coronal sagittal	oblique axial oblique coronal sagittal
FOV(mm)	200	250
Slice thickness(mm)	3.0	3.0
TR(ms)	4200	5780
TE(ms)	101	78
Matrix	320 × 320	320 × 320

*T2WI* T2-weighted imaging, *DWI* Diffusion-weighted imaging, *FOV* Field of view, *TR* Repetition time, *TE* Echo time

### Reference standard for lymphovascular invasion

The reference standard for LVI of rectal cancer was the presence of tumor cells in lymphatic vessels and/or blood vessels in the resected specimen by postoperative pathology. The surface of tumor cells is covered with endothelial cells or thrombosis is found in blood vessels.

### Image interpretation and segmentation

**Fig. 2:**
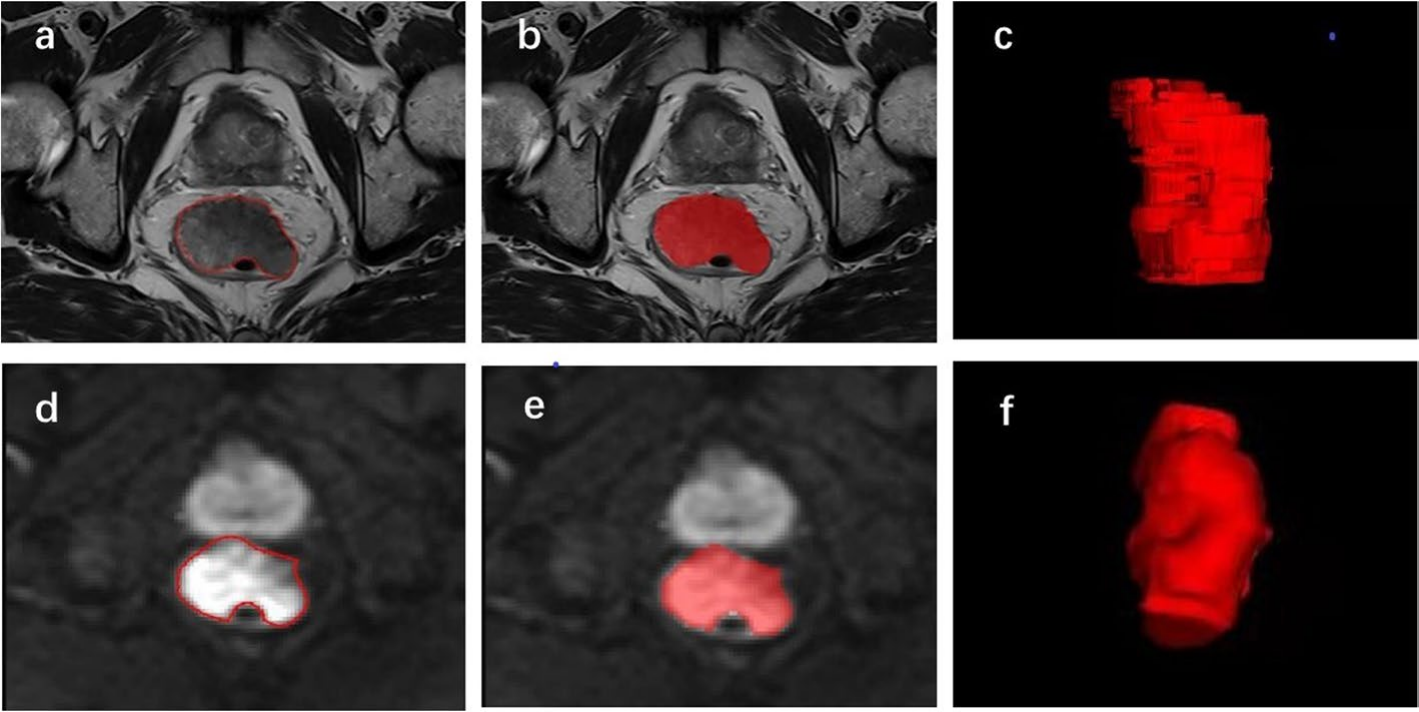
Example of segmentation. Images from a 73-year-old man with rectal adenocarcinoma and pathologically proven lymphovascular invasion. **a**, **b** Delineation of the tumor on T2-weighted images. **d**, **e** Delineation of the tumor on diffusion-weighted images. **c**, **f** Three-dimensional rendering of segmentation of tumor

The patients were randomly divided into a training group (*n* = 102) and a validation group (*n* = 44) at a proportion of 7:3. Previous research has established that the signal intensity of rectal adenocarcinoma on T2WI is higher than that of the muscularis propria but lower than that of the submucosa. On high b-value DWI, the foci of the lesions show significantly higher signal intensity than the normal rectal wall, and the corresponding apparent diffusion coefficient map shows significantly lower signal intensity [15]. In the present study, a physician who had specialized in abdominal imaging for 8 years (reader1) used ITK-SNAP software (version 3.8.0, http://www.itksnap.org/pmwiki/pmwiki.php?n=Main.Publications) to evaluate T2WI and DWI of all planes and delineate the regions of interest on oblique axial images independently. The region of the tumor was identified with reference to the relevant imaging diagnostic criteria [16–18]. The whole-volume three-dimensional delineation method was used in this process as shown in Fig. 2. After one week, 30 cases were randomly selected and delineated by reader 1 and another physician with 12 years of diagnostic experience (reader 2), respectively, after training. The delineation interval between T2WI and DWI of the same patient was more than one day.

### Radiomics feature extraction and model construction

The radiomics features were extracted using PyRadiomics software version 3.0.1 (https://pyradiomics.readthedocs.io/en/latest/index.html) [19], which met the criteria of the Image Biomarker Standardization Initiative (https://theibsi.github.io). The first-order features, shape-based features, and texture features based on Gray-level co-occurrence matrix(GLCM), Gray-level run-length matrix (GLRLM), Gray-level size zone matrix(GLSZM) and Gray-level dependence matrix(GLDM) were extracted, except for the original image.The texture features are also described as the second-order features that describe the intensity level of the spatial distribution of voxels.We also transformed the image using the Laplacian of Gaussian (sigma: 2, 3, 4, 5) and wavelet transformations. In total, 1218 radiomics features were obtained for each image. The default MRI extraction settings provided by PyRadiomics were adopted to extract the features. The basic settings were as follows: resample pixel spacing: 1 × 1 × 1, bin width: 5, normalize: true, and normalize scale: 100. Further details are provided in the Supplemental materials 1 and 2.

The radiomics features of 30 cases delineated by 2 physicians were calculated, and the intra-observer and inter-observer reproducibility of the radiomics features were evaluated. The radiomics features with poor reproducibility should he ruled out.We then used two feature selection methods, minimum redundancy maximum relevance (mRMR) and least absolute shrinkage and selection operator (LASSO), to select the features. First, mRMR was performed to eliminate the redundant and irrelevant features, and 30 features were retained. LASSO was then conducted to choose the optimized subset of features to construct the final model. LASSO involved choosing the regular parameter λ and determining the number of features. After the number of features had been determined, the most predictive subset of features was chosen and the corresponding coefficients were evaluated.

The radiomics score (Radscore) was calculated by summing the selected features weighted by their coefficients using the following formula: $\mathrm y^\wedge=\mathrm b1\mathrm X1+\mathrm b2\mathrm X2+\cdots+\mathrm{biXi}+\mathrm b$, where y^ is the Radscore, b is the intercept, bi is the coefficient of the feature i, and Xi is the value of the feature i. In this way, the Radscore was calculated by summing the selected features weighted by their coefficients.

### Statistical analysis

All statistical analyses were performed with R software version 4.0.3 (https://cran.r-project.org). Continuous variables are expressed as mean and standard deviation, and categorical variables are expressed as frequency and percentage. The t test or rank sum test was used to analyze differences between groups of continuous variables, the chi-square test or Fisher’s test was used for unordered categorical variables, and the rank sum test was used for ordered categorical variables. The intraclass correlation coefficients (ICCs) was used to assess the intra-observer and inter-observer reproducibility of extraction. The ICCs < 0.7 was considered to have poor reproducibility. LASSO regression was used to select radiomics feature parameters, and logistics regression was used to construct model equations. The receiver operating characteristic (ROC) Curves and the area under the receiver operating characteristics curve (AUC) and DeLong’s test were used to evaluate the predictive performance among different models. A *P*-value of < 0.05 was considered statistically significant.

## Results

In total, 146 cases of rectal cancer were included in this study (102 cases in the training group, 44 cases in the validation group). Of these 146 cases, 38 were positive for lymphovascular invasion. There were no significant differences in the clinical or pathological results between the groups. The details are shown in Table 2.

**Table 2:** Clinical and pathological characteristics of patients in training and validation groups

Characteristics	Training group *n* = 102	Validation group *n* = 44	*P* value
Gender,No.(%)			0.217
Male	71 (69.6)	26 (59.1)	
Female	31 (30.4)	18 (40.9)	
Age(mean years,SD)	62.96 (11.78)	62.32 (12.67)	0.768
Location, No.(%)			0.954
high	35 (34.3)	14 (31.8)	
middle	44 (43.1)	20 (45.5)	
low	23 (22.6)	10 (22.7)	
Grade, No.(%)			0.876
Well	14 (13.7)	6 (13.6)	
Moderate	75 (73.5)	31 (70.5)	
Poor	13 (12.8)	7 (15.9)	
pT stage, No.(%)			0.747
T1-2	32 (31.4)	15 (34.1)	
T3a-b	70 (68.6)	29 (65.9)	
pN stage, No.(%)			0.886
N0	52 (51.0)	23 (52.3)	
N1a-b	50 (49.0)	21 (47.7)	
Lymphovascular invasion, No.(%)			0.853
positive	27 (26.5)	11 (25.0)	
negtive	75 (73.5)	33 (75.0)	

Unless otherwise stated, data are presented as number of patients and percentages are shown in parentheses. *SD* Standard deviation, *pT stage* Pathology-based T stage, *pN stage* pathology-based N stage

**Fig. 3:**
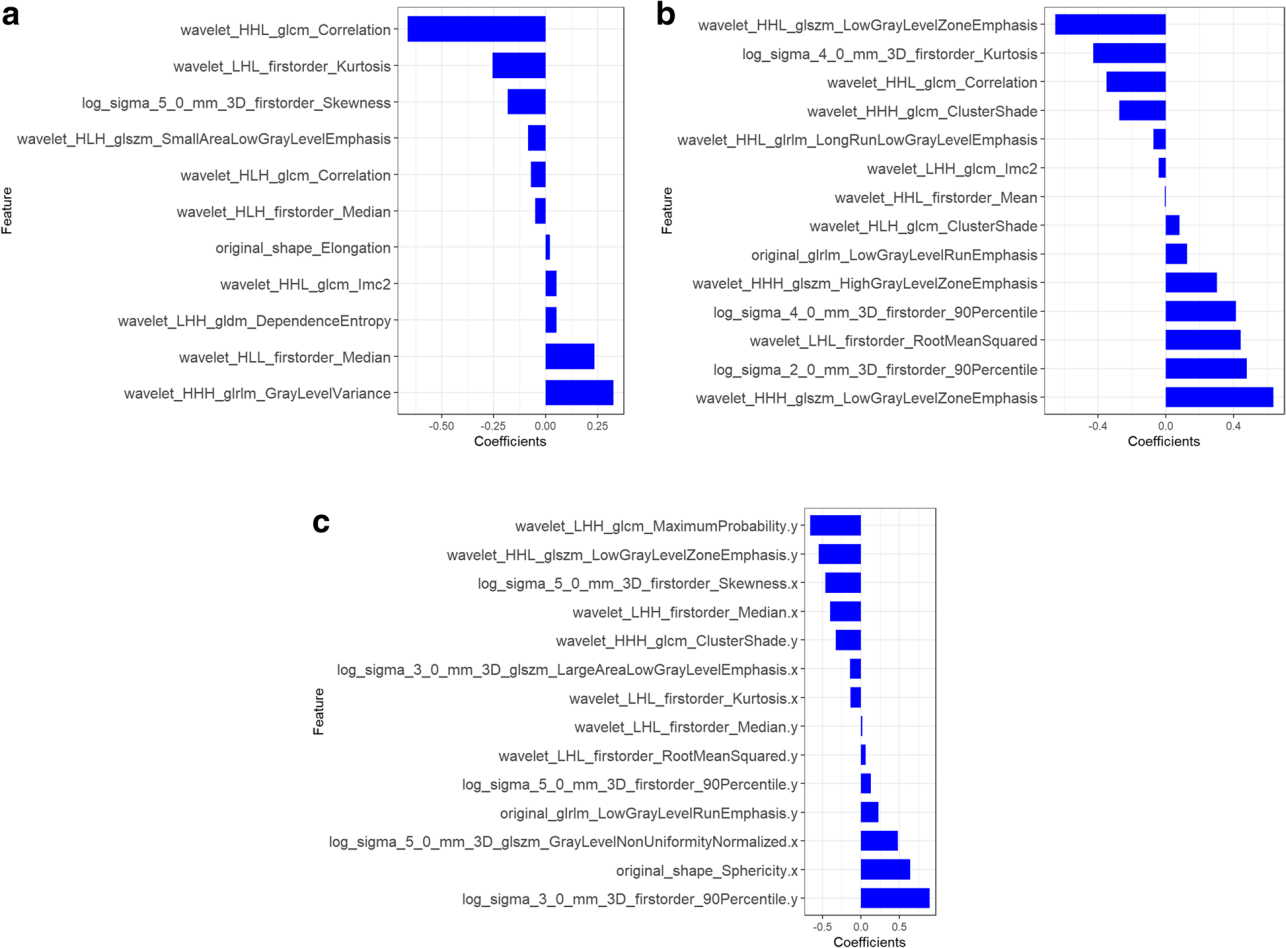
Final radiomics features derived from **a** T2-weighted imaging model, **b** diffusion-weighted imaging model, and **c** combined model

The ICCs of the intra-observer and inter-observer reproducibility were satisfactory. Finally,11 and 14 radiomics features were retained from the T2WI and DWI sequences, respectively. 14 characteristic parameters were retained from the combined model. The details are shown in Fig. 3. The Radscore equations were included in Supplemental materials 1 and 2.

**Fig. 4:**
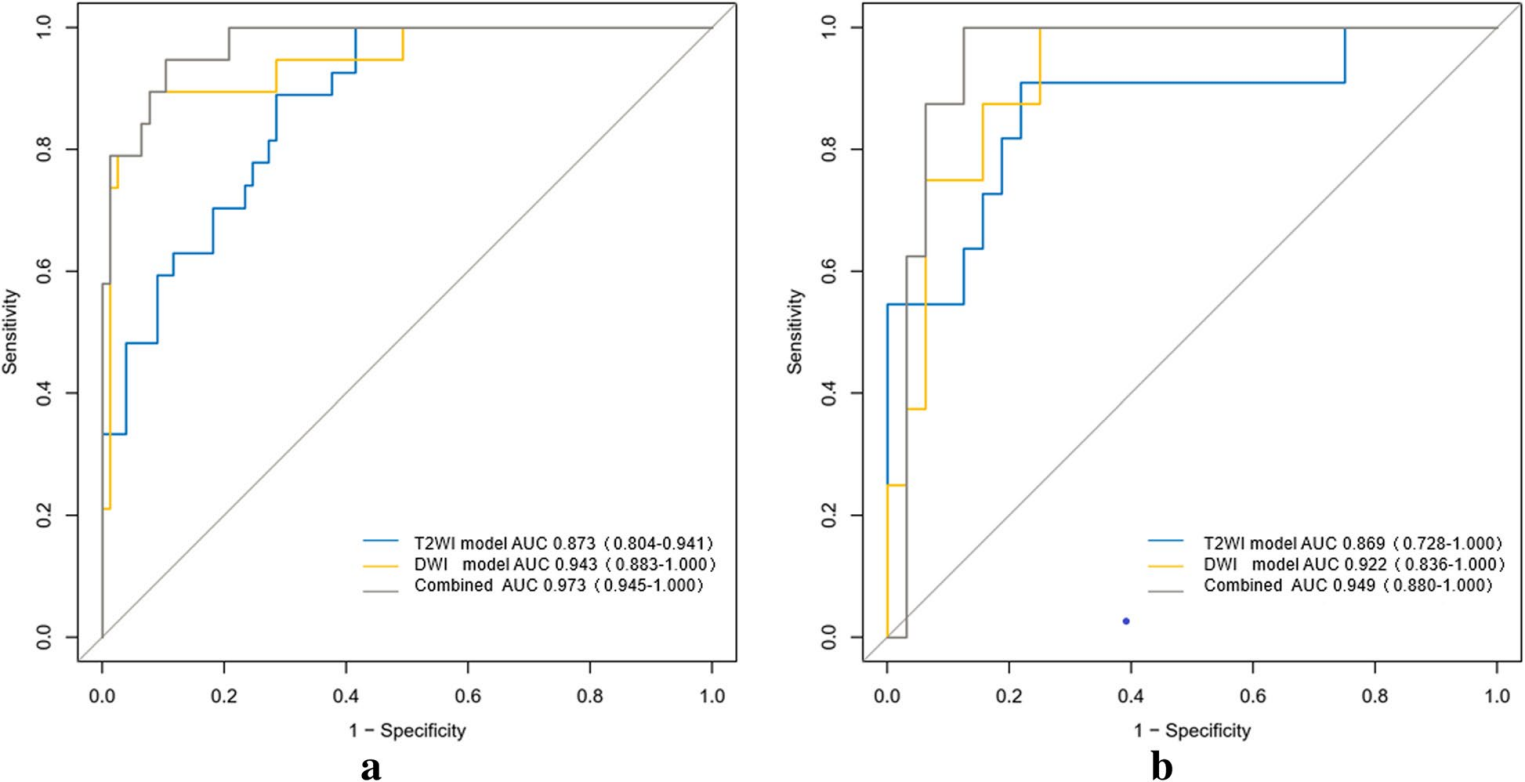
Predictive performance of different models in the training (**a**) and validation (**b**) groups. AUC, area under the receiver operating characteristics curve; T2WI, T2-weighted imaging; DWI, diffusion-weighted imaging

**Fig. 5:**
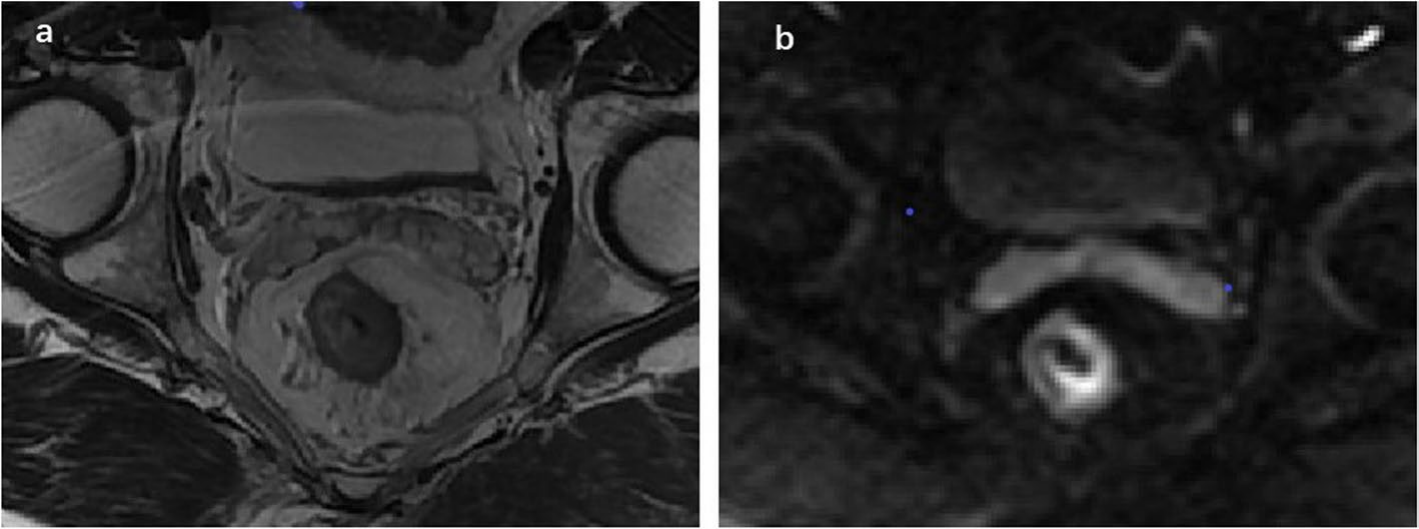
One case of a 73-year-old man with T2N0M0 rectal adenocarcinoma. T2WI(**a**) and DWI(**b**) demonstrated mrEMVI(-). Lymphovascular invasion was detected by DWI model and combined model,which was proved at histopathological examination after surgery

**Fig. 6:**
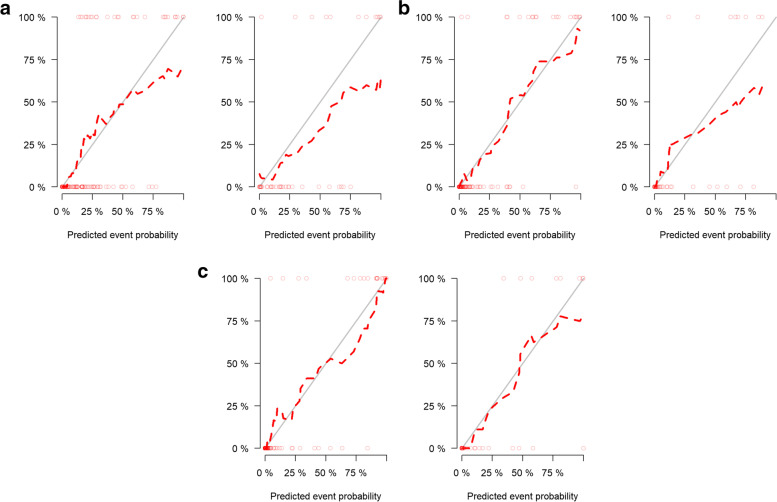
The calibration curves of different models in training and validation groups based on T2WI (**a**),DWI (**b**) and the combined model (**c**)

**Fig. 7:**
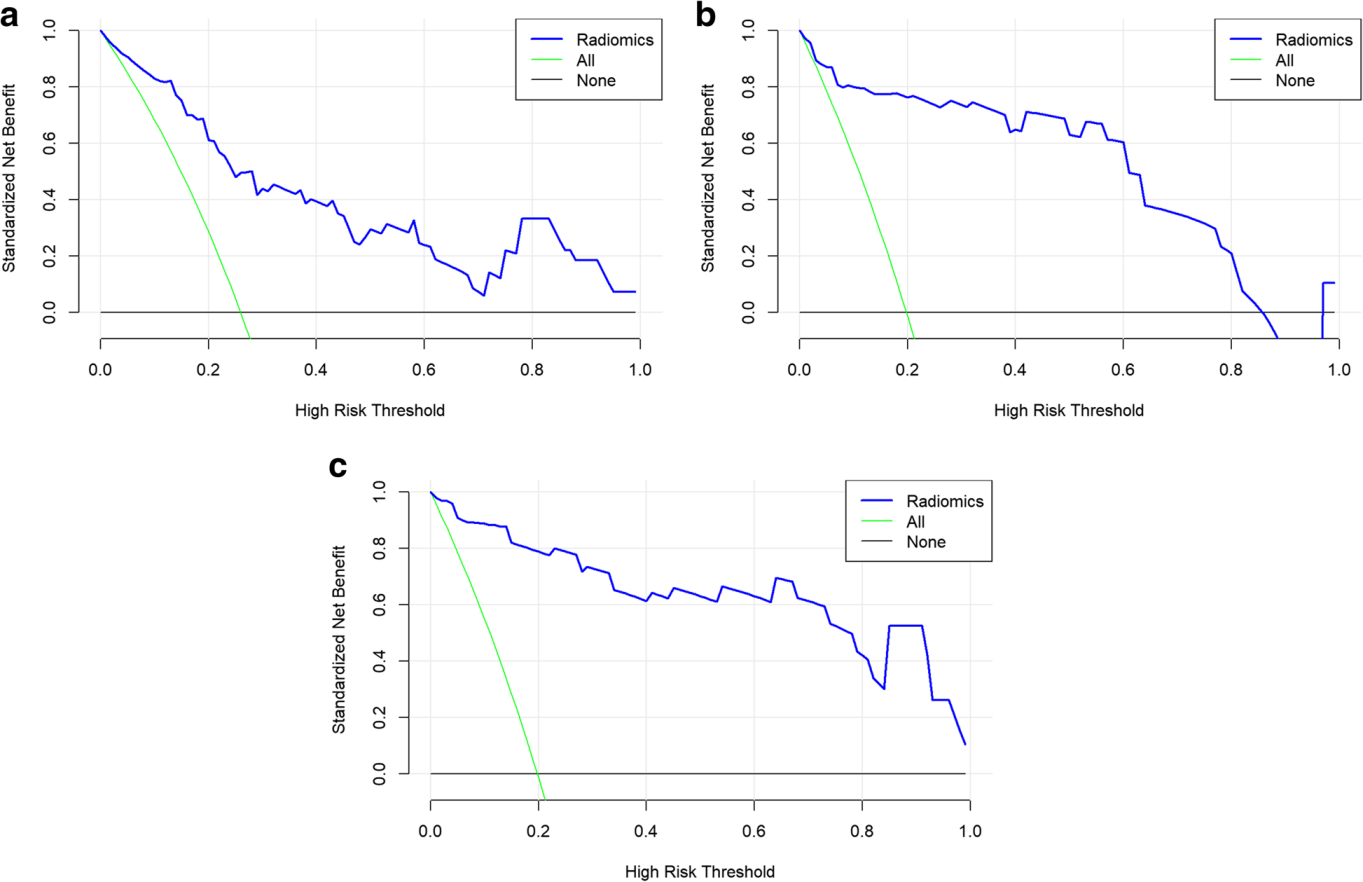
The decision curves analysis of different models in validation group based on T2WI (**a**), DWI (**b**) and the combined model (**c**)

The receiver operating characteristics curve showed that the radiomics model based on T2WI and DWI had good predictive performance for lymphovascular invasion of rectal cancer in both the training cohort and the validation cohort. The AUCs of the T2WI model were 0.87 and 0.87 and the AUCs of the DWI model were 0.94 and 0.92. DeLong’s test showed that the DWI model had better performance than the T2WI model (*P* < 0.05) and that the combined model had better performance than the T2WI model, with AUCs of 0.97 and 0.95 in the training cohort and validation cohort, respectively (*P* < 0.05). The DWI model had predictive performance comparable to that of the combined model (*P* > 0.05) (Table 3, Fig. 4). There was an example as shown in Fig. 5. The calibration curves of the combined model demonstrated the best agreement with the ideal curve ( Fig. 6). The decision curves analysis showed that the combined model had the highest standardized net benefit across the range of 0.1–1.0 of the high risk threshold( Fig. 7).

**Table 3:** Predictive performance of different models in training and validation groups

		Training group				Validation group			
Models	AUC (95%CI)	Accuracy	Sensitivity	Specificity	AUC (95%CI)	Accuracy	Sensitivity	Specificity	cutoff
T2WI model	0.873 (0.804–0.941)	0.760	0.889	0.714	0.869 (0.728–1.00)	0.721	0.909	0.656	-1.12
DWI model	0.943 (0.883–1.000)	0.917	0.895	0.922	0.922 (0.836–1.000)	0.850	0.875	0.844	-0.749
Combined model	0.973 (0.945–1.000)	0.906	0.947	0.896	0.949 (0.880–1.000)	0.875	1.000	0.781	-1.26

*AUC* Area under the receiver operating characteristics curve, *CI* Confidence interval, *T2WI* T2-weighted imaging, *DWI* Diffusion-weighted imaging

## Discussion

High-resolution MRI is currently the main imaging modality for preoperative and postoperative evaluation of patients with rectal cancer, but it cannot be used to obtain a clear diagnosis of small lymphovascular invasion. The present study was performed to explore the preoperative predictive value of biparametric MRI based radiomics features for LVI of rectal cancer, and the results showed that the radiomics models based on T2WI and DWI had good performance. A recent study of rectal cancer showed that a multiparameter radiomics model had good performance in predicting extramural venous invasion of rectal cancer [20]. In the present study, pathologically confirmed cases of LVI were included. Therefore, the evaluation of microcirculation invasion of rectal cancer was more comprehensively in our study. We selected biparametric images, including those obtained from T2WI and DWI sequences, to delineate lesions in this study for several reasons. On the one hand, the biparametric images are easier to obtain clinically than enhanced images with lower economic cost and a shorter inspection time, which is helpful for multicenter research. Without intravenous injection of gadolinium contrast agent, there is no potential risk of residual contrast agent in the body [21, 22]. On the other hand, previous studies have shown that enhanced T1WI has a limited role in staging of rectal cancer and cannot effectively distinguish the signal intensity of the lesions from that of the rectal wall [23, 24]. T2WI and DWI are the most important sequences for the imaging staging of rectal cancer and are effective in demonstrating the lesions [18, 25]. In addition, the whole-tumor three-dimensional method of delineation was used in this study for two reasons. First, because of the limited resolution of MRI, we could not identify the small-diameter arteriovenous and lymphatic vessels on the images directly. It was difficult to accurately delineate. Second, the three-dimensional method can more accurately and fully characterize the voxel characteristics than the two-dimensional method, as shown in previous studies [26, 27]. In recent years, there have been several studies similarly exploring the role of MRI based radiomics for LVI in patients with rectal cancer [28, 29].But differently, we focused on those patients with negative statue of mrEMVI. As the previous evidence showed, MRI has high specificity but moderate sensitivity in the detection of EMVI. There is no need for patients with positive statue of mrEMVI on the basis of routine MRI evaluation to offer extra imaging method for prediction of lymphovasular invasion.

In this study, the results showed that the AUCs of the radiomics models based on T2WI and DWI in the training cohort were 0.87 and 0.87, and those in the validation cohort were 0.94 and 0.92. The AUCs of the combined model were 0.97 and 0.95 in the training cohort and validation cohort, respectively.The results were better than previous study mentioned above.The probable reasons were as follows. First, the patients in our cohort were all evaluated as mrEMVI(-) preoperately. Consequently, the ratio of early stage patients was higher in our study compared with the previous studies. The cystic and necrotic components in early stage rectal cancer are less than advanced stage.Second, we delineated on biparametric MR images rather than multiparametric or multimodal images,which made the process easier and avoid the possibility of making mistakes in delineation on enhamced images.Third, we ruled out the cases of rectal mucinous adenocarcinoma. Rectal mucinous adenocarcinoma is a rare subtype of rectal adenocarcinoma characterized by lakes of stromal mucin containing scant malignant epithelial cells [30, 31]. Thus, it is difficult to identify the solid component on images. The extent of the tumor can be overestimated. As a result, the delineation of the lesions in our study was easier and more accurate.

Moreover, the discrimination of each model was compared. The results showed that the combined model was better than the T2WI model. Interestingly, the AUC of the DWI model was higher than that of the T2WI model and comparable to that of the combined model. As we know, DWI can detect the motion of water molecules in the extracellular and intracellular spaces in vivo, which reflects the cell membrane integrity and cell density [32]. Previous studies have shown that high b-value DWI can improve the accuracy of imaging staging of rectal cancer and has a role in distinguishing tumor foci and fibrous reactions after neoadjuvant chemoradiotherapy [33–35]. In rectal adenocarcinoma, although the component of necrosis and cysts can increase the diffusion, the solid component consisting of the tumor cells always causes diffusion to become restricted. Consequently, the region of tumor cells demonstrates much higher signal intensity than the rectal wall on DWI, which is helpful in detecting the tumor regions and facilitating more accurate delineation. Additionally, the regions of interest on DWI may better characterize tumors and include more biological information than T2WI because DWI has the capability to reflect the tumor microenvironment. Furthermore, the calibration and clinical applicability of the models were taken into consideration. The results showed the combined model demonstrated the best degree of calibration and clinical applicability. The results showed that the combined model can effectively predict LVI of rectal cancer in patients with the mrEMVI (-). DWI was helpful in discriminating the status of LVI in this study.

This study had some limitations. First, the sample size was not large enough. Large-sample multicenter studies are still needed. Second, patients who underwent preoperative neoadjuvant radiotherapy and chemotherapy were excluded. There will be selection bias in this cohort of patients.Third, because of the limited sample size, cases of vascular and lymphatic invasion were not separately divided into groups. Fourth, this was a retrospective study. Finally, the patients’ clinicopathological data was not included in the predictive model.

In conclusion, the radiomics model based on bipapametric MRI showed good preoperative predictive value for lymphovascular invasion of rectal cancer with the mrEMVI (-). Radiomics from bipapametric MRI has the potential to serve as a noninvasive and effective biomarker to predict the status of lymphovascular invasion in rectal cancer preoperatively.

## Supplementary Information

**Additional file 1.**

**Additional file 2.**

## Data Availability

The datasets used and/or analysed during the current study are available from the corresponding author on reasonable request.

## Abbreviations

AUC: Area under the receiver operating characteristics curve
CI: Confidence interval
DWI: Diffusion-weighted imaging
FOV: Field of view
GLCM: Gray-level co-occurrence matrix
GLRLM: Gray-level run-length matrix
GLSZM: Gray-level size zone matrix
GLDM: Gray-level dependence matrix
ICCs: Intraclass correlation coefficients
LASSO: Least absolute shrinkage and selection operator
LVI: Lymphovascular invasion
MRI: Magnetic resonance imaging
mRMR: Minimum redundancy maximum relevance
Radscore: Radiomics score
ROC: Receiver operating characteristic
SD: Standard deviation T2WI:T2-weighted imaging
TR: Repetition time
TE: Echo time
mrEMVI: MRI-based extramural vascular invasion
