# Supplementary material for: Biparametric magnetic resonance imaging-based radiomics features for prediction of lymphovascular invasion in rectal cancer
Source: BMC Cancer. 2023 Jan 18;23:61. doi: 10.1186/s12885-023-10534-w (PMC9847040; doi:10.1186/s12885-023-10534-w)
Supplement: Supplementary file 1 — Additional file 1. [file 12885_2023_10534_MOESM1_ESM.docx]

# This is an example of settings that can be used as a starting point for analyzing MR data with small (~3mm) slice

# thickness. This is only intended as a starting point and is not likely to be the optimal settings for your dataset.

# Some points in determining better values are added as comments where appropriate

# When adapting and using these settings for an analysis, be sure to add the PyRadiomics version used to allow you to

# easily recreate your extraction at a later timepoint:

# ############################# Extracted using PyRadiomics version: <version> ######################################

imageType:

Original: {}

LoG:

# If you include sigma values >5, remember to also increase the padDistance. Because of resampling to (2, 2, 2), the

# use of sigmas < 2 mm is not recommended.

sigma: [2.0, 3.0, 4.0, 5.0]

Wavelet: {}

featureClass:

# redundant Compactness 1, Compactness 2 an Spherical Disproportion features are disabled by default, they can be

# enabled by specifying individual feature names (as is done for glcm) and including them in the list.

shape:

firstorder:

glcm: # Disable SumAverage by specifying all other GLCM features available

- 'Autocorrelation'

- 'JointAverage'

- 'ClusterProminence'

- 'ClusterShade'

- 'ClusterTendency'

- 'Contrast'

- 'Correlation'

- 'DifferenceAverage'

- 'DifferenceEntropy'

- 'DifferenceVariance'

- 'JointEnergy'

- 'JointEntropy'

- 'Imc1'

- 'Imc2'

- 'Idm'

- 'Idmn'

- 'Id'

- 'Idn'

- 'InverseVariance'

- 'MaximumProbability'

- 'SumEntropy'

- 'SumSquares'

glrlm:

glszm:

gldm:

setting:

# Normalization:

# MR signal is usually relative, with large differences between scanners and vendors. By normalizing the image before

# feature calculation, this confounding effect may be reduced. However, if only one specific scanner is used, or the

# images reflect some absolute world value (e.g. ADC maps, T2maps (NOT T2 weighted)), consider disabling the

# normalization.

normalize: true

normalizeScale: 100 # This allows you to use more or less the same bin width.

# Resampling:

# If slices are very thin (~1mm), such as in 3D scanned (isotropic) volumes, resampledPixelSpacing may be reduced to

# (1, 1, 1). Furthermore, in case of isotropic volumes, consider disabling resampling.

# On a side note: increasing the resampled spacing forces PyRadiomics to look at more coarse textures, which may or

# may not increase accuracy and stability of your extracted features.

interpolator: 'sitkBSpline'

resampledPixelSpacing: [1, 1, 1]

# Mask validation:

# correctMask and geometryTolerance are not needed, as both image and mask are resampled, if you expect very small

# masks, consider to enable a size constraint by uncommenting settings below:

#minimumROIDimensions: 2

#minimumROISize: 50

# Image discretization:

# The ideal number of bins is somewhere in the order of 16-128 bins. A possible way to define a good binwidt is to

# extract firstorder:Range from the dataset to analyze, and choose a binwidth so, that range/binwidth remains approximately

# in this range of bins.

binWidth: 5

# first order specific settings:

# When normalizing, gray values below the mean will be negative. Shifting by 300 (3 StdDevs * 100) ensures that the

# majority of voxels is positive (only outliers >3 SD lower than the mean will be negative).

voxelArrayShift: 300

# Misc:

# default label value. Labels can also be defined in the call to featureextractor.execute, as a commandline argument,

# or in a column "Label" in the input csv (batchprocessing)

label: 1
