# Supplementary material for: Biparametric magnetic resonance imaging-based radiomics features for prediction of lymphovascular invasion in rectal cancer
Source: BMC Cancer. 2023 Jan 18;23:61. doi: 10.1186/s12885-023-10534-w (PMC9847040; doi:10.1186/s12885-023-10534-w)
Supplement: Supplementary file 2 — Additional file 2. [file 12885_2023_10534_MOESM2_ESM.docx]

The final equations of radscore is

Radscore=-0.663*wavelet_HHL_glcm_Correlation+-0.049*wavelet_HLH_firstorder_Median+0.052*wavelet_HHL_glcm_Imc2+-0.256*wavelet_LHL_firstorder_Kurtosis+0.02*original_shape_Elongation+0.327*wavelet_HHH_glrlm_GrayLevelVariance+-0.083*wavelet_HLH_glszm_SmallAreaLowGrayLevelEmphasis+-0.071*wavelet_HLH_glcm_Correlation+0.234*wavelet_HLL_firstorder_Median+0.054*wavelet_LHH_gldm_DependenceEntropy+-0.182*log_sigma_5_0_mm_3D_firstorder_Skewness + -1.271" **（T2WI）**

Radscore=0.442*wavelet_LHL_firstorder_RootMeanSquared+0.48*log_sigma_2_0_mm_3D_firstorder_90Percentile+0.304*wavelet_HHH_glszm_HighGrayLevelZoneEmphasis+0.125*original_glrlm_LowGrayLevelRunEmphasis+-0.273*wavelet_HHH_glcm_ClusterShade+0.082*wavelet_HLH_glcm_ClusterShade+-0.35*wavelet_HHL_glcm_Correlation+0.637*wavelet_HHH_glszm_LowGrayLevelZoneEmphasis+-0.073*wavelet_HHL_glrlm_LongRunLowGrayLevelEmphasis+-0.654*wavelet_HHL_glszm_LowGrayLevelZoneEmphasis+-0.005*wavelet_HHL_firstorder_Mean+-0.043*wavelet_LHH_glcm_Imc2+0.416*log_sigma_4_0_mm_3D_firstorder_90Percentile+-0.429*log_sigma_4_0_mm_3D_firstorder_Kurtosis + -2.114"**(DWI)**

Radscore=0.059*wavelet_LHL_firstorder_RootMeanSquared.y+-0.547*wavelet_HHL_glszm_LowGrayLevelZoneEmphasis.y+0.13*log_sigma_5_0_mm_3D_firstorder_90Percentile.y+0.644*original_shape_Sphericity.x+-0.658*wavelet_LHH_glcm_MaximumProbability.y+0.48*log_sigma_5_0_mm_3D_glszm_GrayLevelNonUniformityNormalized.x+0.226*original_glrlm_LowGrayLevelRunEmphasis.y+-0.46*log_sigma_5_0_mm_3D_firstorder_Skewness.x+-0.323*wavelet_HHH_glcm_ClusterShade.y+-0.401*wavelet_LHH_firstorder_Median.x+0.895*log_sigma_3_0_mm_3D_firstorder_90Percentile.y+-0.14*log_sigma_3_0_mm_3D_glszm_LargeAreaLowGrayLevelEmphasis.x+-0.137*wavelet_LHL_firstorder_Kurtosis.x+0.022*wavelet_LHL_firstorder_Median.y + -2.229" **（combined）**
